# Conservative Management With Tranexamic Acid for Intrahepatic Pseudoaneurysm After Unsuccessful Biliary Drainage in a Patient on Apixaban

**DOI:** 10.7759/cureus.106005

**Published:** 2026-03-27

**Authors:** Pouyan Golshani, Andrew K Song, Hoon Kang, Maya Singh

**Affiliations:** 1 Interventional Radiology, Kaiser Permanente Los Angeles Medical Center, Los Angeles, USA; 2 Interventional Radiology, Kaiser Permanente Riverside Medical Center, Riverside, USA; 3 Molecular And Cell Biology, University of California, Berkeley, Berkeley, USA

**Keywords:** anticoagulant-related bleeding, apixaban and aspirin, biliary intervention, common hepatic artery pseudoaneurysm, conservative medical management, hepatic hemorrhage, interventional radiology guided embolization, oral tranexamic acid, pseudoaneurysm thrombosis

## Abstract

Conservative management with tranexamic acid (TXA) is known in difficult-to-access pseudoaneurysms, including those of the pulmonary artery, and its broad efficacy is seen in hemorrhagic pathologies such as subarachnoid hemorrhage. We present the case of a 90-year-old woman with paroxysmal atrial fibrillation on apixaban, remote cholecystectomy, and multiple comorbidities (including hypothyroidism, interstitial lung disease, and asthma) who was admitted for right upper quadrant pain, nausea, and laboratory evidence of cholestasis (total bilirubin 3.0 mg/dL, aspartate aminotransferase 250 U/L). Computed tomography was suggestive of choledocholithiasis, and gastroenterology recommended briefly holding apixaban before endoscopic retrograde cholangiopancreatography (ERCP). On hospital day 3, ERCP was abandoned because of an upper esophageal sphincter narrowing and Zenker’s diverticulum. Interventional radiology then performed a percutaneous transhepatic cholangiography with a 22-gauge Chiba needle, but nondilated intrahepatic ducts prevented drain placement. Ultimately, a laparoscopic-assisted ERCP achieved successful common bile duct stone extraction. Postprocedurally, the patient’s hemoglobin declined from 13.6 to 9.4 g/dL (without transfusions), and abdominal imaging identified a 1.8-cm intrahepatic pseudoaneurysm plus a 4.1-cm peripancreatic hematoma. Diagnostic angiography demonstrated delayed pseudoaneurysm filling without a definable feeding artery suitable for embolization. Given the small lesion and occult arterial source, we administered oral TXA 1,000 mg three times daily to promote thrombosis. Within 48 hours, hemoglobin stabilized around 8.8 g/dL, and repeat CT revealed spontaneous pseudoaneurysm thrombosis. The patient was discharged uneventfully with stable hemoglobin and had no pseudoaneurysm reaccumulation at short-term follow-up. Should TXA have failed, we had discussed a potential percutaneous thrombin injection, which has shown success for endovascularly inaccessible hepatic pseudoaneurysms. This case underscores the risk of iatrogenic bleeding in patients on direct oral anticoagulants who require multiple biliary interventions and highlights how a short course of TXA can be a practical alternative when standard embolization is not feasible.

## Introduction

Intrahepatic pseudoaneurysms are rare but potentially life-threatening complications of hepatobiliary trauma and intervention, including liver biopsy and biliary drainage. Delayed rupture can result in significant hemorrhage if not promptly recognized. Endovascular embolization is the standard treatment; however, this approach depends on the identification of a discrete arterial feeder and may not be feasible in small, intraparenchymal, or angiographically occult lesions. When embolization is not technically achievable, management options are limited, and there is little guidance regarding conservative treatment in hemodynamically stable patients without active extravasation [1].

Tranexamic acid (TXA), an antifibrinolytic agent widely used in trauma and perioperative bleeding, has been reported in limited vascular settings, including pulmonary artery pseudoaneurysms and intracranial hemorrhage [2,3]. Alternative minimally invasive strategies, such as percutaneous thrombin injection, have also been described [4]. TXA is a synthetic lysine analogue that reduces fibrinolysis and stabilizes clots [5]. More recently, large randomized trials such as POISE-3 (PeriOperative ISchemic Evaluation-3) have further characterized the bleeding and thrombotic risk profile of TXA in high-risk patients [6]. A 2013 meta-analysis demonstrated a reduction in rebleeding with antifibrinolytic therapy, without a corresponding reduction in mortality [7].

In this context, we describe the conservative use of oral TXA in an elderly anticoagulated patient with an angiographically occult intrahepatic pseudoaneurysm following unsuccessful biliary drainage, adding to the limited clinical experience with non-interventional management strategies in select cases.

## Case presentation

A 90-year-old woman with paroxysmal atrial fibrillation on apixaban, hypothyroidism, interstitial lung disease, and asthma presented with right upper quadrant pain, nausea, and cholestatic liver enzyme elevation (total bilirubin 3.0 mg/dL, aspartate aminotransferase (AST) 250 U/L).

Computed tomography (CT) of the abdomen revealed choledocholithiasis. Apixaban was held, and endoscopic retrograde cholangiopancreatography (ERCP) was attempted but aborted due to an upper esophageal sphincter narrowing and a Zenker’s diverticulum. Interventional radiology performed a percutaneous transhepatic cholangiography (PTC) using a 22-gauge Chiba needle, but nondilated intrahepatic ducts prevented drain placement. A subsequent laparoscopic-assisted ERCP successfully removed the common bile duct stones.

**Figure 1:**
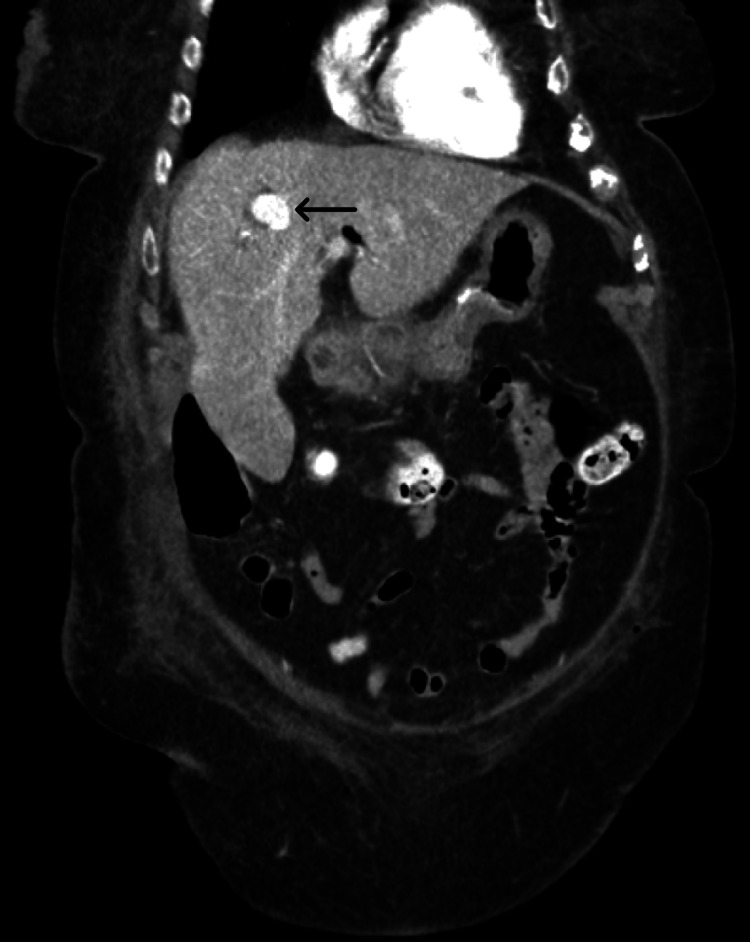
Coronal CT image showing a 1.8-cm intrahepatic pseudoaneurysm (arrow) in the right hepatic lobe.

**Figure 2:**
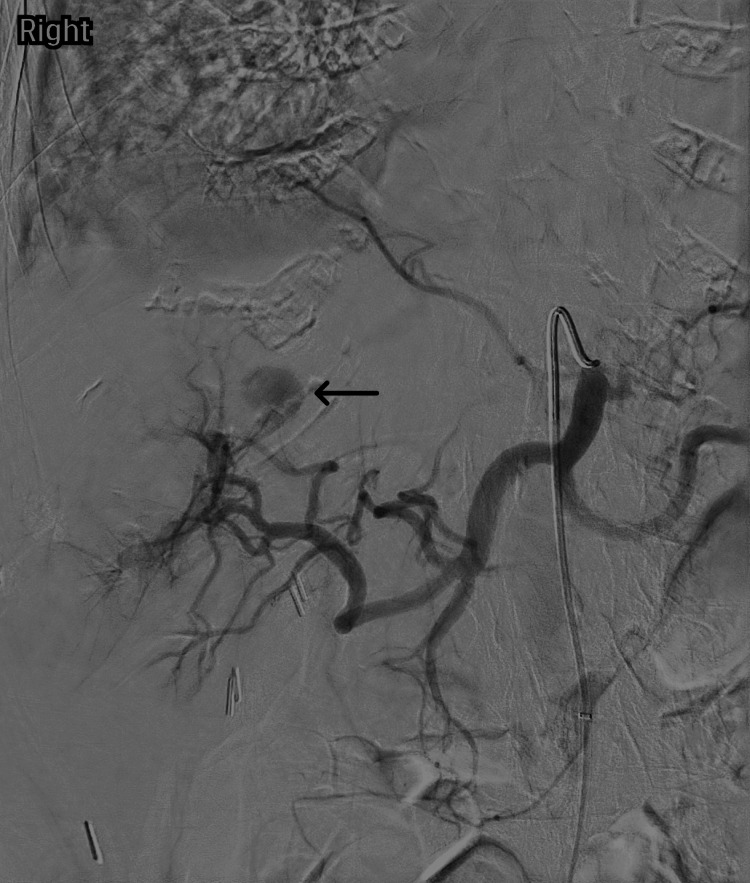
Delayed angiographic phase demonstrating faint filling of the pseudoaneurysm (arrow) and no discrete feeding vessel.

Post procedure, the patient’s hemoglobin declined from 13.6 g/dL to 9.4 g/dL (without transfusion). CT imaging identified a 1.8-cm intrahepatic pseudoaneurysm and a 4.1-cm peripancreatic hematoma (Figure 1). The patient subsequently underwent super-selective interrogation of the right hepatic artery and segmental/subsegmental branches using a 2.8-F Progreat microcatheter (Terumo Corporation, Tokyo, Japan) over a preloaded microwire, but no discrete feeding artery suitable for safe embolization was identified. Angiography demonstrated delayed pseudoaneurysm filling (Figure 2). Given the small lesion and occult arterial source, oral TXA 1,000 mg three times daily was initiated to promote thrombosis. 

**Figure 3:**
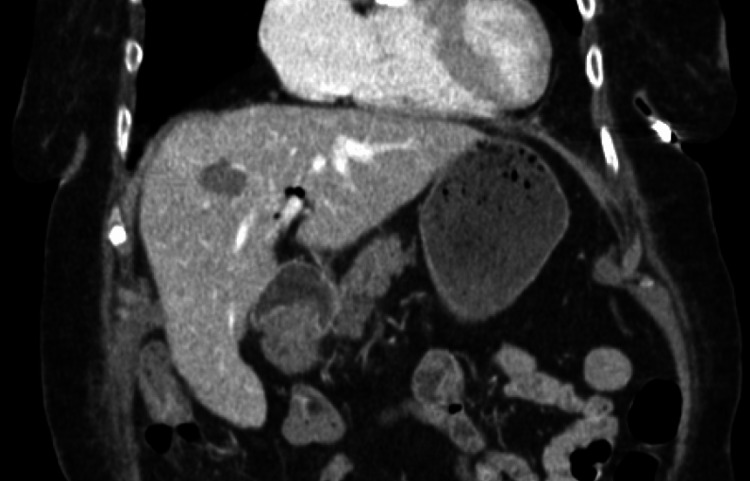
Follow-up CT after two days of tranexamic acid therapy revealing pseudoaneurysm thrombosis and stable peripancreatic hematoma

Within 48 hours, hemoglobin stabilized around 8.8 g/dL, and repeat CT demonstrated complete pseudoaneurysm thrombosis with a stable peripancreatic hematoma (Figure 3). Furthermore, there were no percutaneous/anatomic limitations that would have precluded escalation to percutaneous therapy; percutaneous thrombin injection was considered as a backup option if the patient destabilized or the pseudoaneurysm progressed. The patient was discharged uneventfully with no pseudoaneurysm recurrence at short-term follow-up.

## Discussion

Hepatic pseudoaneurysms most commonly arise following trauma, biliary intervention, or liver biopsy. Conventional management involves endovascular embolization; however, some pseudoaneurysms, particularly small or intraparenchymal lesions, may lack a clearly identifiable feeding vessel, rendering embolization technically unfeasible.

TXA has been reported in the management of other vascular bleeding conditions, including pulmonary artery pseudoaneurysms [2] and subarachnoid hemorrhage [3], where it is used to limit fibrinolysis and promote clot stability. TXA is included in the World Health Organization Model List of Essential Medicines [8] and is widely utilized in settings such as trauma, surgical hemorrhage, and perioperative bleeding. Its established safety profile and oral formulation make it a potential consideration in select cases of contained hemorrhage when procedural options are limited. Additionally, the POISE-3 trial demonstrated that TXA reduces bleeding risk in patients undergoing noncardiac surgery without a significant increase in major thrombotic events, further supporting its overall hemostatic safety in appropriately selected patients [6].

In the present case, TXA administration was temporally associated with clinical stabilization and imaging-confirmed thrombosis of the pseudoaneurysm within 48 hours, without observed adverse events. TXA is a synthetic lysine analogue that competitively inhibits the activation of plasminogen to plasmin, thereby reducing fibrinolysis and stabilizing existing clots [5]. While this mechanism supports its use in hemorrhagic conditions, a causal relationship between TXA administration and pseudoaneurysm thrombosis cannot be established from a single case.

Although selective and superselective right hepatic arteriography was performed, the pseudoaneurysm opacified only on delayed runs, and no discrete embolizable feeding vessel could be identified. Given the absence of a safely targetable feeder, empiric segmental embolization was deferred due to non-target ischemia risk in an elderly patient with a small, clinically contained lesion; percutaneous thrombin injection remained a contingency option if clinical or imaging progression occurred.

Minimally invasive strategies, such as percutaneous thrombin injection [4], have been described for pseudoaneurysms that are inaccessible to embolization. In select, hemodynamically stable patients without evidence of active extravasation, TXA may represent a conservative adjunct or temporizing measure when both endovascular and percutaneous interventions are not feasible. TXA is presented as a conservative adjunct in carefully selected stable patients when standard endovascular therapy is not technically achievable. As a single-case observation, this report cannot establish causality between TXA administration and pseudoaneurysm thrombosis, and further studies are needed to better define its role, safety, and patient selection criteria in the management of intrahepatic pseudoaneurysms.

## Conclusions

In this case, embolization was not technically feasible due to the absence of a safely targetable feeding vessel despite angiographic evaluation, and hence, a short course of oral TXA was used with close clinical and imaging follow-up. Interval CT demonstrated pseudoaneurysm thrombosis with hemoglobin stabilization. However, this is a single-case observation and does not establish a causal relationship between TXA administration and pseudoaneurysm thrombosis.

Endovascular embolization remains first-line therapy for hepatic pseudoaneurysms when a target vessel can be identified. TXA should be considered only as a conservative adjunct in carefully selected, hemodynamically stable patients when definitive intervention is not technically achievable, with a low threshold to escalate to endovascular or percutaneous therapy if there is clinical deterioration or imaging progression.
